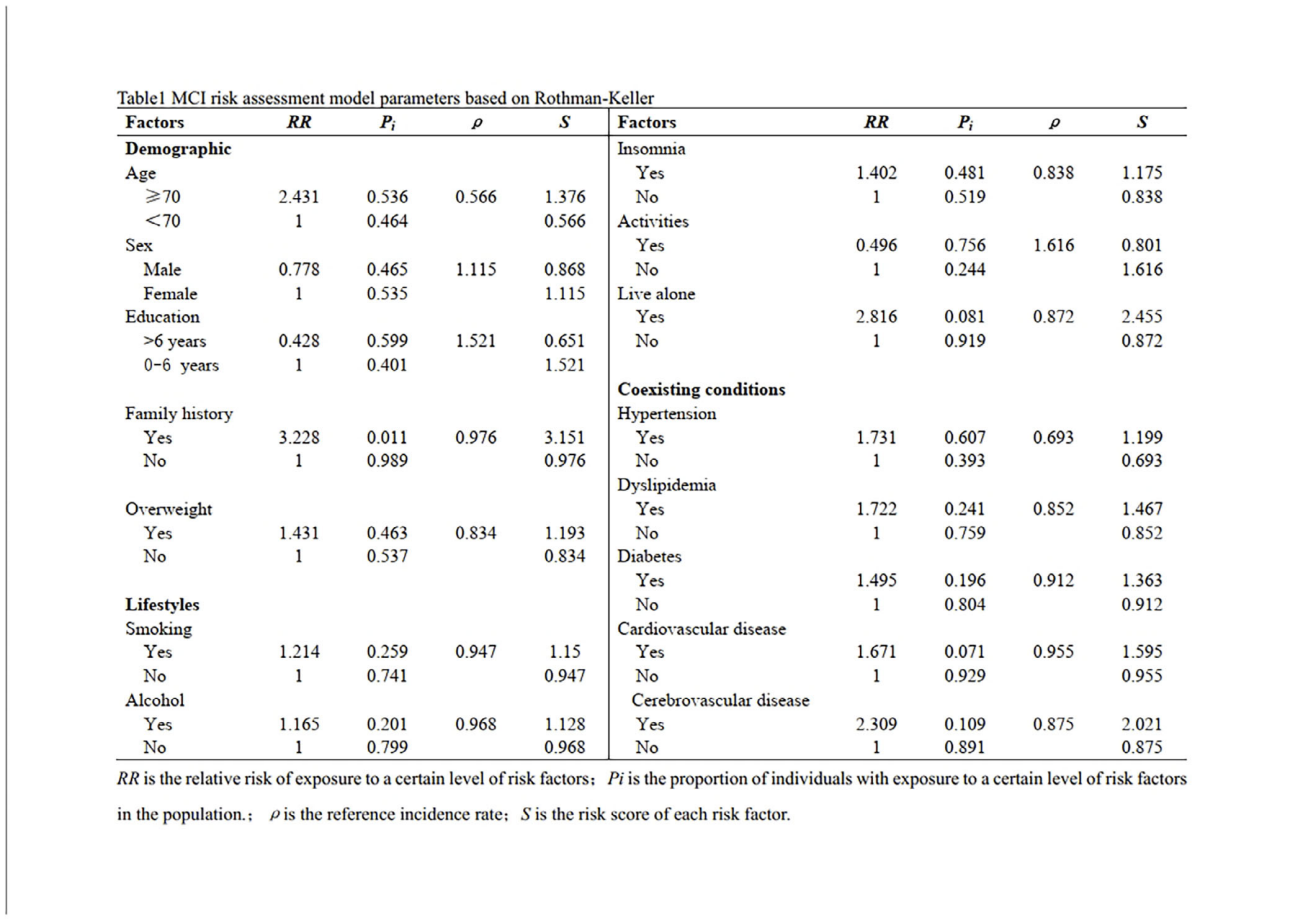# Establishment of a Practical Risk Assessment Model for Mild Cognitive Impairment (MCI) in the Elderly

**DOI:** 10.1002/alz.086033

**Published:** 2025-01-09

**Authors:** Jinlei Li, Gongwu Ding

**Affiliations:** ^1^ Peking Union Medical College, Beijing, Beijing China

## Abstract

**Background:**

With a rapidly aging population, general practitioners (GPs) in communities are confronting the challenge of determining those who are at greatest risk for cognitive impairment and potentially need more specialized follow‐up and intervention to mitigate symptoms early in their course. Mild cognitive impairment (MCI) serves as an important stage for the early intervention and prevention of dementia. However, most MCI survey questionnaires require heavy investments of manpower and time, which limits the possibility of their application among GPs in communities. Therefore, we aimed to build and validate an MCI risk assessment model using potential predictors that are readily available to GPs, to identify high‐risk populations and further offer targeted cognitive impairment screening in communities.

**Method:**

The study retrieved MCI risk factors for the elderly and then conducts quantitative analysis for the retrieved information via meta‐analysis, to determine generally available risk factors and their level of significance to MCI. The Rothman‐Keller risk assessment model was constructed based on risk factors, which takes into consideration not only the independent effects of the influencing factors but also their interactions. The performance of the Rothman‐Keller model was evaluated using the ROC curve using data from 2,545 seniors in communities.

**Result:**

A total of 49 papers were retrieved for meta‐analysis and 15 risk factors were finally included in the MCI risk assessment model. The AUC of the MCI risk assessment model established in this study was 0.772 (95%CI: 0.753∼0.791), with a sensitivity of 78.04% and specificity of 63.95%. We also further formulate a tool (applet) that can be used in primary care, to identify with a high‐risk MCI population and delay the occurrence of dementia in communities based on the risk assessment model.

**Conclusion:**

This model not only affords GPs the opportunity to conduct cognitive primary screening in the clinic setting but also helps individuals to identify their potential risk profile and trigger interventions that may delay or even prevent progression to dementia. The public health consequences are to reduce future dementia incidence rates.